# Association of Regular Piano Training with Dental Anxiety and Stress in Children Undergoing Pit and Fissure Sealant Application: A Cross-Sectional Study

**DOI:** 10.3390/children13101367

**Published:** 2026-10-08

**Authors:** Eda Nur Ural, Sema Aydınoğlu, Dilara Nil Günaçar

**Affiliations:** 1 Department of Pediatric Dentistry, Faculty of Dentistry, Recep Tayyip Erdoğan University, Rize 53020, Türkiye; 2 Department of Oral and Maxillofacial, Radiology, Faculty of Dentistry, Recep Tayyip Erdoğan University, Rize 53020, Türkiye

**Keywords:** pediatric dentistry, dental anxiety, stress, music education

## Abstract

**Highlights:**

**What are the main findings?**

- Children with regular piano training showed lower dental anxiety and perceived stress than children without instrumental training.
- Exploratory within-group analyses controlling for age suggested that longer formal piano education was associated with lower perceived stress, while greater weekly formal piano lesson duration was associated with lower post-treatment dental anxiety and perceived stress; these findings were based on *p* values unadjusted for multiple comparisons.

**What are the implications of the main findings?**

- Long-term active musical participation may be associated with lower anxiety and perceived stress responses during pediatric dental care.
- Regular instrumental training may represent a potential psychosocial factor associated with children’s responses during dental treatment.

**Abstract:**

**Background/Objectives:** Regular musical training has been associated with emotional regulation and stress coping, but its relationship with children’s dental responses remains unclear. This study evaluated associations between regular piano training and dental anxiety, perceived stress, and physiological responses during fissure sealant application. **Methods:** Eighty systemically healthy children aged 7–11 years were categorized as a piano group (PG; n = 40) or control group (CG; n = 40) based on pre-existing piano training. All underwent standardized fissure sealant application using Tell–Show–Do. Dental anxiety and perceived stress were assessed before and after treatment using MCDAS_f_, FIS, physiological parameters, and PeSSKi. **Results:** Pre-treatment MCDAS_f_ scores did not differ between groups, whereas post-treatment scores were lower in the PG. Overall FIS scores were lower in the PG. PeSSKi scores were lower in the PG at T1 and remained lower at T2, indicating a between-group difference already present before the dental procedure. Significant group × time interactions were observed for heart rate, systolic blood pressure, and MCDAS_f_. Post-treatment MCDAS_f_, FIS, and PeSSKi differences remained significant after adjustment for baseline scores and parental education. In exploratory within-group analyses controlling for age, longer formal piano education was associated with lower PeSSKi scores at T1 and T2, while greater weekly formal piano lesson duration was associated with lower post-treatment MCDAS_f_ and PeSSKi scores. These *p* values were unadjusted for multiple comparisons and should therefore be interpreted cautiously. **Conclusions:** Regular piano training was associated with lower dental anxiety and perceived stress and selected physiological responses during fissure sealant application.

## 1. Introduction

Dental anxiety is defined as an intense feeling of apprehension associated with dental treatment and is often linked to a perceived loss of control during dental procedures [1]. In pediatric patients, uncontrolled dental anxiety may lead to behavioral management difficulties, postponement or avoidance of dental care, and deterioration of oral health status over time. Furthermore, negative dental experiences during childhood may contribute to the persistence of anxiety-related behaviors into adulthood [2]. Early identification of anxiety levels allows clinicians to anticipate treatment-related difficulties and implement appropriate behavioral management strategies in a timely manner [3].

The assessment of dental anxiety requires a multidimensional approach integrating behavioral observations, psychometric scales, projective techniques, and physiological measurements. Since children may have difficulty verbally expressing emotional states depending on their developmental stage, age-appropriate assessment tools are of particular importance [2]. The Modified Child Dental Anxiety Scale—faces version (MCDAS_f_) is widely used for evaluating dental anxiety in children aged 5–12 years using facial representations [3]. Similarly, the Facial Image Scale (FIS) is considered a reliable projective assessment tool for evaluating emotional responses and anxiety levels in pediatric dental settings [4]. Physiological parameters such as heart rate (HR), blood pressure (BP), peripheral oxygen saturation (SpO_2_), and body temperature (BT) are frequently used as objective indicators of anxiety-related autonomic responses during dental treatment [5].

Stress is defined as a multidimensional process characterized by physiological, psychological, and behavioral responses that emerge when environmental or psychological demands exceed an individual’s coping capacity [6,7]. The Perceived Stress Scale for Kids (PeSSKi) is considered suitable for children aged 7–11 years and evaluates stress-related emotions, thoughts, and experiences. Higher total scores indicate higher levels of perceived stress [8].

Music is regarded as a multidimensional stimulus associated with emotional regulation, stress management, and physiological relaxation processes [9]. Recent evidence suggests that music-based interventions may be associated with lower anxiety and fear in pediatric healthcare settings [10]. Beyond passive music listening, music education and active instrumental training require sustained engagement of auditory, motor, attentional, and cognitive-control processes. In children, structured music training has been associated with cognitive benefits, particularly in executive functions such as inhibitory control, working memory, and cognitive flexibility [11]. Because executive functions contribute to self-regulation, they may provide a plausible link between long-term musical practice and the regulation of emotional and stress responses. Active engagement in music has also been associated with emotion-regulation processes, although the extent to which these findings generalize to children in stressful clinical settings remains unclear [12].

From a neurodevelopmental perspective, long-term musical practice has been associated with experience-dependent neuroplasticity in auditory and sensorimotor systems, with longitudinal evidence supporting training-related plasticity from childhood to adulthood [13]. Piano training is particularly relevant because it requires sustained attention, bimanual coordination, fine motor control, sensorimotor integration, auditory feedback, performance monitoring, and structured repetitive practice. Longitudinal neuroimaging research in novice pianists has demonstrated training-related reorganization of auditory-motor and sensorimotor networks during piano learning [14]. Together, these cognitive, sensorimotor, and neuroplastic characteristics provide a rationale for specifically examining whether regular piano training is associated with children’s self-regulatory and stress-related responses in challenging situations, including dental treatment.

Although music-based interventions have increasingly been used as supportive approaches in pediatric healthcare, previous studies in pediatric dentistry have predominantly focused on short-term passive music-listening interventions administered during dental procedures [15]. Consequently, it remains unclear whether pre-existing long-term piano training is associated with dental anxiety, perceived stress, and anxiety-related physiological responses in children undergoing a standardized dental procedure.

Therefore, this study aimed to examine the association between regular piano training and dental anxiety, perceived stress, and physiological parameters in children undergoing pit and fissure sealant application and to compare these outcomes with those of children without instrumental training.

## 2. Materials and Methods

### 2.1. Study Design and Ethics

This cross-sectional observational study was conducted at the Pediatric Dentistry Clinic, Department of Pediatric Dentistry, Faculty of Dentistry, Recep Tayyip Erdoğan University, Rize, Türkiye. The study protocol was approved by the Non-Interventional Clinical Research Ethics Committee of Recep Tayyip Erdoğan University Faculty of Medicine (31 July 2025; No: 2025/348). Participant enrollment and data collection were conducted between 3 August 2025 and 9 January 2026. The study by Kabasakal et al. [16] was conducted using a separately recruited participant sample, and there was no overlap in participants or participant-level data between that study and the present study.

Although ethical approval was obtained before participant enrollment, the study was retrospectively registered at ClinicalTrials.gov on 20 May 2026 (NCT07602075), after completion of participant enrollment and data collection. This study was conducted in accordance with the principles of the Declaration of Helsinki and its subsequent amendments. As all participants were children aged 7–11 years, their parents or legal guardians received detailed information regarding the study procedures, and written informed consent was obtained before participation.

### 2.2. Sample Size

The sample size was determined a priori using G*Power software (version 3.1.9.7). As no previous study had specifically investigated dental anxiety in children in relation to regular piano or musical instrument training, a directly comparable effect-size estimate was unavailable. Therefore, an expected effect size of Cohen’s d = 0.85 was calculated from the mean and standard deviation values of MCDASf scores reported by Xing et al. [17], an available pediatric study addressing anxiety-related outcomes. With an alpha level of 0.05 and a statistical power of 0.95, a minimum of 37 participants per group was required (total n = 74). Additional power calculations for within-group anxiety and stress outcomes yielded smaller required sample sizes; therefore, 37 participants per group was considered the determining sample size. Considering potential participant loss, the target sample size was increased to 80 children (40 per group).

### 2.3. Inclusion Criteria

Children aged 7–11 years were included if they were systemically healthy (ASA I), had no known allergies or hearing impairments, were cognitively capable of understanding the questionnaires, presented with indications for fissure sealant application on their mandibular permanent first molars, and scored 3 (positive) or 4 (definitely positive) on the Frankl Behavior Rating Scale [18].

### 2.4. Exclusion Criteria

Children with any systemic disease or allergy history, Frankl Behavior Rating Scale scores of 1 or 2, active psychological or psychiatric treatment, or physical or cognitive limitations precluding communication or questionnaire completion were excluded.

### 2.5. Study Procedure

Children attending the pediatric dental clinic and those enrolled in piano courses were invited to participate. After assessment of the eligibility criteria, 60 eligible children with regular piano training for at least 3 months and 60 eligible children without any previous experience of playing a musical instrument constituted the sampling pools for the piano group (PG) and control group (CG), respectively. From each pool, 40 children were randomly selected using a computer-generated random sampling procedure (Randomizer.org), resulting in 80 participants (40 per group). The random selection was performed by an independent clinical nurse who was not involved in the clinical procedures or outcome assessments. Randomization was used solely for participant selection and not for group allocation; group membership was determined by the participants’ pre-existing piano training status.

**Figure 1:**
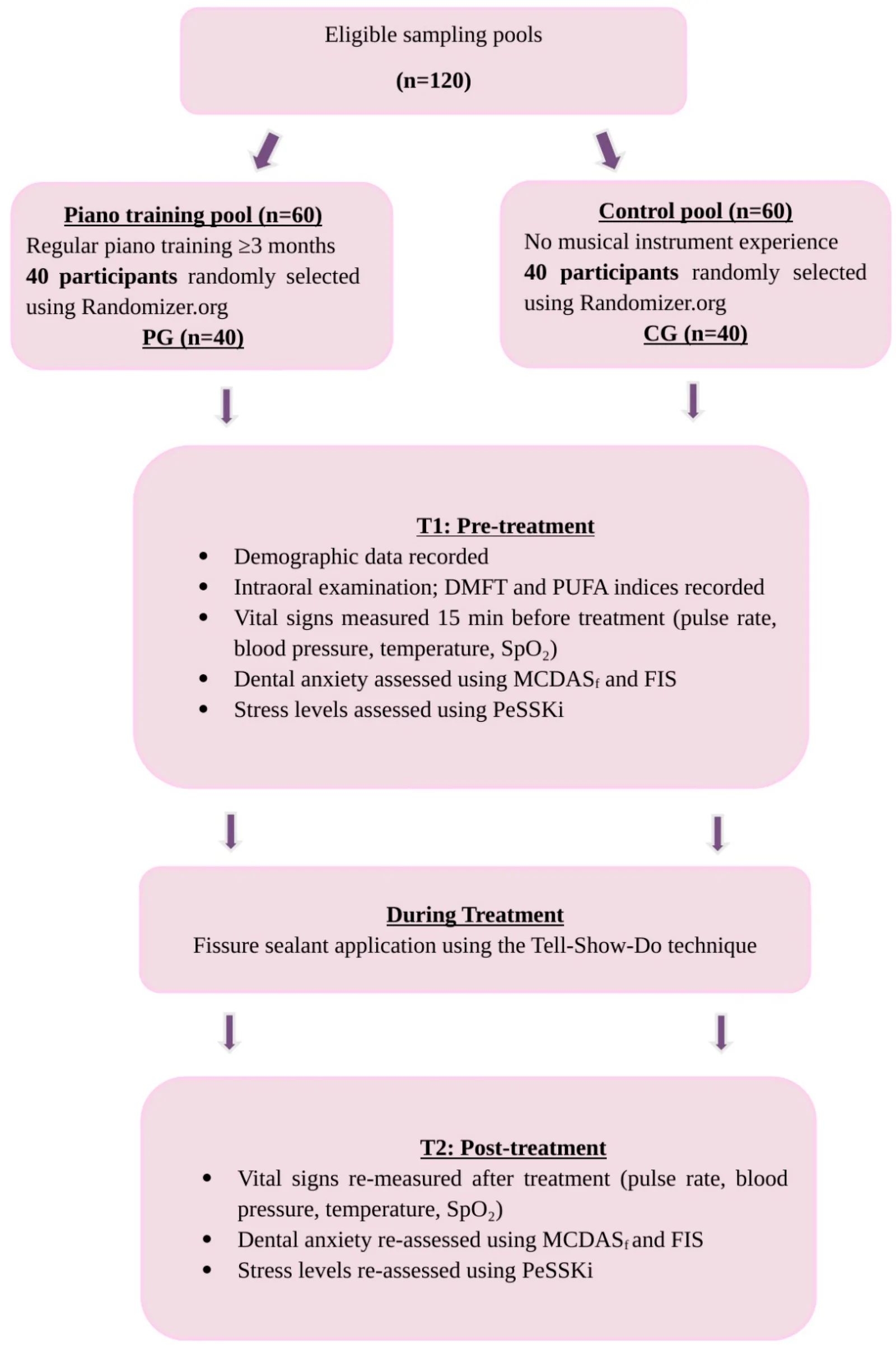
Study flowchart.

For each participant in the PG, the total duration of formal piano education (months), weekly formal piano lesson duration (hours/week), and home piano practice duration outside formal lessons (hours/week) were recorded. These piano-training characteristics were subsequently examined in relation to dental anxiety and perceived stress outcomes within the PG. The overall study flowchart is presented in Figure 1.

Preventive fissure sealant treatment was performed on all participants by the same pediatric dentist (E.N.U.) using a standardized clinical protocol. Approximately 15 min before treatment, the pediatric dentist, S.A. recorded HR, BP, SpO_2_, and BT while the children were seated in the waiting area. Participants were then seated in the dental unit, and an intraoral examination was performed. Dental caries experience was assessed using the Decayed, Missing, and Filled Teeth (DMFT) index according to World Health Organization criteria [19]. The clinical consequences of untreated dental caries were assessed using the PUFA index developed by Monse et al. [20], which records visible pulpal involvement (P), ulceration caused by dislocated tooth fragments (U), fistula (F), and abscess (A). The MCDAS_f_, FIS, and PeSSKi were subsequently administered by the pediatric dentist, S.A., who had 15 years of clinical experience and was independent of the clinical procedure. The pre-treatment assessments were recorded as T1.

Before fissure sealant application, the procedure and instruments were explained and demonstrated to all participants in an age-appropriate manner using the Tell–Show–Do technique [21]. The explanation and familiarization period was standardized to approximately 10 min to support psychological preparation and ensure consistent behavior guidance. Treatment was then performed according to a standardized protocol. The mandibular permanent first molars were polished to remove plaque and debris, and isolation was achieved using cotton rolls and a saliva ejector. The enamel surface was etched with phosphoric acid gel (RubyEtch Etching Gel, İnci Dental, Istanbul, Türkiye) for 15 s according to the manufacturer’s instructions. The surface was then rinsed and air-dried for approximately 10 s until a chalky white enamel appearance was obtained. A resin-based fissure sealant (Ruby Pit and Fissure Sealant, İnci Dental, Istanbul, Türkiye) was applied to the pits and fissures and distributed using a micro-applicator. No separate bonding agent was used. The sealant was polymerized for 20 s using an LED curing unit (LED.G, Guilin Woodpecker Medical Instrument Co., Ltd. Guilin, China). Retention was checked using a dental explorer, and occlusion was verified using articulating paper. All clinical steps were completed within a standardized 10 min treatment session for each participant.

Immediately after treatment, physiological parameters were measured again, and the MCDAS_f_, FIS, and PeSSKi were re-administered by the pediatric dentist, S.A.; these post-treatment assessments were recorded as T2. To minimize environmental factors that could influence children’s anxiety and stress responses, all procedures were carried out in the same clinical setting under standardized conditions, without parental presence.

### 2.6. Outcome Measures

#### 2.6.1. Evaluation of Dental Anxiety

Dental anxiety assessment in children should consider age, cognitive development, and emotional expression; therefore, psychometric, projective, and physiological methods may be used together [22]. In the present study, dental anxiety was evaluated using the MCDAS_f_ as a psychometric assessment tool, the FIS as a projective assessment tool, and physiological indicators. The MCDAS_f_ is a valid and reliable scale developed to assess dental anxiety in children aged 5–12 years using facial representations [3]. The FIS is a projective tool that enables children to express their current emotional state through facial images and is used for the assessment of dental anxiety in pediatric dental settings [4]. In addition, physiological parameters, including HR, BP, SpO_2_, and BT were recorded as objective physiological measures potentially associated with anxiety-related responses.

#### 2.6.2. Psychometric Evaluation

Dental anxiety was psychometrically assessed using the MCDAS_f_, an eight-item scale in which each item is scored from 1 (not anxious) to 5 (extremely anxious), yielding a total score ranging from 8 to 40; higher scores indicate greater dental anxiety [3]. Each item was explained to the child, and the child was asked to select the facial expression that best represented their emotional state. To ensure standardization, the pediatric dentist, S.A. was trained to provide the same verbal instructions to all participants during scale administration. The MCDAS_f_ was administered before treatment (T1) and immediately after treatment (T2). The Turkish version of the MCDAS_f_ has been demonstrated to be valid and reliable [23].

#### 2.6.3. Projective Evaluation

Dental anxiety was assessed using the FIS, a five-point facial expression scale ranging from 1 (very happy) to 5 (very unhappy), with higher scores indicating a more negative emotional state and increased dental anxiety. During administration, children were asked to select the facial image that best represented how they felt at that moment, and the selected score was recorded. Buchanan and Niven reported that the FIS is a valid tool for assessing child dental anxiety status in a clinical context [4]. The FIS was administered by the pediatric dentist, S.A. before treatment (T1) and immediately after treatment (T2).

#### 2.6.4. Physiological Evaluation

Physiological parameters, including BP, HR, BT, and SpO_2_, were measured by the pediatric dentist, S.A. approximately 15 min before treatment while the children were seated in the waiting area (T1) and immediately after treatment while they were seated in the dental unit (T2).

#### 2.6.5. Blood Pressure (BP) and Heart Rate (HR)

Systolic and diastolic BP and HR were measured simultaneously using a child-appropriate digital wrist blood pressure monitor (Polygreen, K-Jump Health Co., Ltd., New Taipei City, Taiwan) while the child was seated comfortably in a relaxed position.

#### 2.6.6. Peripheral Oxygen Saturation (SpO_2_)

SpO_2_ was measured using a fingertip pulse oximeter (Artı Med, Model C03, Istanbul, Türkiye). Measurements were recorded while the child was seated comfortably, with the sensor placed on the index finger.

#### 2.6.7. Body Temperature (BT)

BT was measured using a non-contact infrared thermometer (Nimomed, Hünkâr Ecza ve Medikal San. ve Tic. Ltd. Şti., Istanbul, Türkiye). Measurements were recorded from the forehead while the child was seated comfortably.

#### 2.6.8. Evaluation of Perceived Stress

Perceived stress was evaluated using the PeSSKi. The PeSSKi is a 10-item Likert-type scale used to assess perceived stress levels in children aged 7–11 years. The scale consists of two subdimensions: perceived self-efficacy and perceived helplessness. Children were asked to select the most appropriate response for each item, and the total score was recorded. Higher total scores indicate higher levels of perceived stress [8]. The Turkish version of the PeSSKi has been demonstrated to be valid and reliable [24]. The PeSSKi was administered before treatment (T1) and immediately after treatment (T2).

### 2.7. Statistical Analysis

Statistical analyses were performed by an independent statistician from eİstatistik (Atakum, Samsun, Türkiye) using R version 4.5.0 and IBM SPSS Statistics version 23. The normality of continuous variables was assessed using the Shapiro–Wilk test. Descriptive data were presented as mean ± standard deviation (SD) or median (minimum–maximum) for continuous variables, and as frequency and percentage for categorical variables. Between-group comparisons of non-normally distributed continuous variables were performed using the Mann–Whitney U test. Associations between categorical variables were analyzed using Fisher’s exact test, Yates’ correction, or Monte Carlo-corrected Fisher’s exact test, as appropriate. Multiple comparisons for categorical variables were performed using the Bonferroni-corrected Z test.

For MCDAS_f_, FIS, and PeSSKi scores, generalized estimating equations (GEE) were used to account for the within-subject correlation arising from repeated measurements. Participant ID was specified as the subject variable, and an exchangeable working correlation structure was assumed. The models included Group, Time, and Parental Education as main effects, together with the Group × Time interaction. Where appropriate, pairwise comparisons based on estimated marginal means were performed with Bonferroni adjustment for multiple comparisons.

To further evaluate between-group differences in post-treatment MCDAS_f_, FIS, and PeSSKi scores while accounting for pre-treatment scores and parental education, additional ANCOVA models were performed. For each psychological outcome, the post-treatment (T2) score was entered as the dependent variable, Group and Parental Education were included as fixed factors, and the corresponding pre-treatment (T1) score was entered as a covariate. Before ANCOVA, the assumptions of normality, homogeneity of variances, homogeneity of regression slopes, and linearity were assessed. Standard ANCOVA was used when the relevant assumptions were satisfied. When the normality assumption was violated, bootstrap ANCOVA with 500 bootstrap samples was applied, whereas HC3 heteroskedasticity-consistent robust ANCOVA was used when the homogeneity of variance assumption was not met. Partial eta squared (partial η^2^) with 95% confidence intervals (CIs) was reported as the effect-size measure for the ANCOVA models.

As exploratory analyses, associations between piano training characteristics (duration of formal piano education, weekly formal piano lesson duration, and home piano practice duration outside formal lessons) and MCDAS_f_, FIS, and PeSSKi scores at T1 and T2 within the PG were evaluated using partial correlation analyses controlling for age. No adjustment for multiple comparisons was applied to these 18 exploratory within-group correlations; therefore, the resulting *p* values should be interpreted as unadjusted and hypothesis-generating.

For physiological outcomes, normally distributed repeated-measures data were analyzed using GEE with Bonferroni-adjusted post hoc comparisons, whereas non-normally distributed repeated-measures data were analyzed using aligned rank transform (ART) mixed ANOVA with Holm correction for multiple comparisons. Statistical significance was set at *p* < 0.05.

## 3. Results

A total of 80 children were included in the study, with 40 participants in the PG and 40 in the CG. All 80 participants completed the planned fissure sealant procedure and both T1 and T2 assessments; no participant withdrew or was excluded after treatment initiation, and no treatment was discontinued. No significant differences were observed between the groups in terms of age, sex, family income level, history of previous dental visits, DMFT index, or PUFA index (*p* > 0.05). However, parental education level differed significantly between the groups (*p* = 0.001), with a higher proportion of university-educated parents in the PG (90%) than in the CG (55%).

GEE analyses of MCDAS_f_, FIS, and PeSSKi scores are presented in Table 1. For MCDAS_f_, significant main effects of Group (*p* = 0.008) and Time (*p* < 0.001), as well as a significant Group × Time interaction (*p* < 0.001), were observed. Overall MCDAS_f_ scores were lower in the PG than in the CG (17.71 ± 4.68 vs. 19.59 ± 3.89). MCDAS_f_ scores decreased from 20.40 ± 4.35 at T1 to 15.03 ± 3.27 at T2 in the PG and from 21.18 ± 3.39 to 18.00 ± 3.74 in the CG. Post hoc comparisons indicated a greater reduction in MCDAS_f_ scores in the PG. Parental Education was not significantly associated with MCDAS_f_ scores in the GEE model (*p* = 0.318). For FIS, significant main effects of Group (*p* = 0.017) and Time (*p* < 0.001) were observed. Overall FIS scores were lower in the PG than in the CG (1.50 ± 0.69 vs. 1.78 ± 0.69), and scores decreased from T1 to T2 across groups (2.01 ± 0.72 vs. 1.26 ± 0.44). Parental Education was not significantly associated with FIS scores (*p* = 0.594), and the Group × Time interaction was not significant (*p* = 0.531). For PeSSKi, significant main effects of Group and Time were observed (both *p* < 0.001). Overall PeSSKi scores were lower in the PG than in the CG (19.78 ± 5.34 vs. 30.59 ± 3.33), and scores decreased from T1 to T2 across groups (26.13 ± 6.89 vs. 24.24 ± 7.04). Neither Parental Education (*p* = 0.070) nor the Group × Time interaction (*p* = 0.597) was statistically significant. Descriptive statistics for MCDAS_f_, FIS, and PeSSKi scores according to parental education level are presented in Supplementary Table S1.

Additional baseline-adjusted analyses were performed to examine whether between-group differences in post-treatment scores persisted after accounting for the corresponding pre-treatment scores and parental education (Table 2). After adjustment for the corresponding pre-treatment score and parental education, significant between-group differences remained for post-treatment MCDAS_f_ scores (*p* < 0.001, partial η^2^ = 0.319, 95% CI: 0.140–0.518), FIS scores (*p* = 0.020, partial η^2^ = 0.073, 95% CI: 0.002–0.240), and PeSSKi scores (*p* = 0.042, partial η^2^ = 0.059, 95% CI: 0.001–0.236). Descriptive statistics for post-treatment MCDAS_f_, FIS, and PeSSKi scores according to group and parental education level are provided in Supplementary Table S2.

Parental education was significantly associated with post-treatment MCDAS_f_ scores (*p* = 0.003, partial η^2^ = 0.112, 95% CI: 0.023–0.273) and PeSSKi scores (*p* = 0.024, partial η^2^ = 0.055, 95% CI: 0.001–0.210) but not with post-treatment FIS scores (*p* = 0.355, partial η^2^ = 0.017, 95% CI: 0.000–0.140). The corresponding pre-treatment score was significantly associated with the post-treatment score for MCDAS_f_ (*p* < 0.001), FIS (*p* = 0.014), and PeSSKi (*p* < 0.001).

The comparison of physiological parameters according to group and time is presented in Table 3. HR values showed significant effects of time (*p* = 0.026) and group × time interaction (*p* < 0.001), whereas the overall group effect was not statistically significant (*p* = 0.783). HR decreased from T1 to T2 in the PG, whereas a slight increase was observed in the CG. For systolic BP, neither the overall group effect (*p* = 0.406) nor the time effect (*p* = 0.097) was statistically significant. However, a significant group × time interaction was observed (*p* = 0.028), with systolic BP decreasing after treatment in the PG, whereas no significant change was observed in the CG. For SpO_2_, a significant effect of time was observed (*p* = 0.032), whereas the overall group effect and group × time interaction were not statistically significant. No statistically significant group × time interactions were observed for diastolic BP or BT (both *p* > 0.05).

In the PG, the mean duration of formal piano education was 23.15 ± 14.13 months, with a median duration of 24 months (range: 3–72 months). The mean weekly formal piano lesson duration was 1.15 ± 0.36 h/week, with a median of 1 h/week (range: 1–2), while the mean home piano practice duration outside formal lessons was 1.63 ± 0.89 h/week, with a median of 1.75 h/week (range: 0–4). The correlations between piano training characteristics and anxiety and stress measures are presented in Table 4. After controlling for age, duration of formal piano education was significantly negatively associated with PeSSKi scores at T1 (r = −0.354, *p* = 0.027) and T2 (r = −0.376, *p* = 0.018), whereas its association with post-treatment MCDAS_f_ did not reach statistical significance (r = −0.315, *p* = 0.051). Weekly formal piano lesson duration was significantly negatively associated with post-treatment MCDAS_f_ (r = −0.461, *p* = 0.003) and PeSSKi scores (r = −0.419, *p* = 0.008). No significant associations were observed between home piano practice duration and any of the psychological outcomes, and no significant associations were observed between FIS scores and any of the piano training characteristics.

## 4. Discussion

Dental anxiety is a multifactorial condition that may negatively affect treatment compliance in children and influence long-term oral health behaviors [1,2]. Although music-based approaches have increasingly been investigated in pediatric dentistry, previous studies have predominantly focused on passive music listening during dental treatment rather than on pre-existing, long-term active instrumental training [15]. Therefore, this study evaluated the association between regular piano training and psychological and physiological responses during pit and fissure sealant application in children.

Our primary findings showed that children in the PG had lower MCDAS_f_, FIS, and PeSSKi scores than those in the CG. Significant group × time interactions were observed for MCDAS_f_ scores, HR, and systolic BP, whereas no significant group × time interactions were found for FIS, PeSSKi, SpO_2_, diastolic BP, or BT. Importantly, baseline-adjusted analyses showed that between-group differences in post-treatment MCDAS_f_, FIS, and PeSSKi scores remained significant after accounting for the corresponding pre-treatment scores and parental education. Within the PG, exploratory analyses controlling for age showed that longer duration of formal piano education was associated with lower PeSSKi scores at both T1 and T2, while greater weekly formal piano lesson duration was associated with lower post-treatment MCDAS_f_ and PeSSKi scores. Overall, regular piano training was associated with lower dental anxiety and perceived stress, while selected piano training characteristics were negatively associated with specific anxiety and stress measures within the PG. Differences in patterns of change were also observed for selected physiological parameters.

The similarity between the groups in terms of age, sex, family income, previous dental experience, DMFT, and PUFA scores indicates comparability with respect to these measured characteristics; however, it does not exclude the possibility of residual confounding. Dental anxiety in children has been reported to be influenced by individual factors such as age, sex, and previous dental experiences [25]. However, the higher parental education level observed in the PG is particularly relevant when interpreting the present findings. Parental education may be associated not only with children’s access to sustained extracurricular activities such as music lessons, but also with health literacy, educational opportunities, behavioral development, and family-related coping resources [25,26]. Given this between-group imbalance, parental education was included in the adjusted analyses. In the GEE models, parental education was not significantly associated with FIS, MCDAS_f_, or PeSSKi scores, while the main effect of Group remained significant for all three outcomes. In the baseline-adjusted ANCOVA models, however, parental education was significantly associated with post-treatment MCDAS_f_ and PeSSKi scores, but not with FIS. The differing findings regarding parental education may be related to differences in model specification, as the GEE models assessed outcomes across the repeated T1 and T2 measurements, whereas the ANCOVA models evaluated post-treatment scores after adjustment for the corresponding baseline scores. Nevertheless, between-group differences in post-treatment MCDAS_f_, FIS, and PeSSKi scores remained significant after adjustment for both the corresponding pre-treatment scores and parental education. Parental education represents only one dimension of the broader socioeconomic and sociocultural context. Although family income did not differ significantly between the groups, other unmeasured family-level factors may still have contributed to the observed associations. Therefore, residual confounding cannot be entirely excluded.

Janthasila and Keeratisiroj [27] evaluated children aged 10–12 years undergoing fissure sealant treatment and reported lower dental anxiety and fear scores following music therapy and aromatherapy, suggesting that non-pharmacological approaches may be beneficial for anxiety management even during preventive dental procedures. A systematic review and meta-analysis of non-pharmacological behavioral interventions in children younger than 12 years reported that distraction techniques were associated with lower self-reported anxiety, better cooperation, and lower pulse rates than the Tell–Show–Do technique, except during more invasive dental procedures. In contrast, findings regarding virtual reality, modeling, visual pedagogies, Tell–Show–Do, and other non-pharmacological approaches were inconsistent [28]. The review also highlighted substantial heterogeneity across studies in terms of age groups, dental procedures, intervention methods, and anxiety assessment tools [28]. These findings underscore the importance of considering both developmental stage and procedural characteristics when comparing pediatric dental anxiety studies. Accordingly, the present findings should be interpreted specifically within the context of 7–11-year-old children undergoing pit and fissure sealant application.

In addition, all participants were systemically healthy and had Frankl Behavior Rating Scale scores of 3 or 4, corresponding to positive or definitely positive behavior [18]. The inclusion of systemically healthy children provided a more homogeneous sample by reducing the potential influence of systemic conditions or related medications on psychological and physiological responses; however, it also limits the generalizability of the findings to children with systemic diseases or special healthcare needs. Lazarova and Mitova similarly evaluated children with Frankl scores of 3 or 4 and reported that cooperative behavior does not necessarily indicate the absence of underlying dental anxiety [29]. Thus, although the present eligibility criteria ensured a relatively cooperative clinical sample, Frankl scores of 3–4 primarily reflect observable behavior and do not, by themselves, exclude dental anxiety. Accordingly, the present findings may not be directly generalizable to children with negative or definitely negative behavior (Frankl scores 1–2).

The lower MCDAS_f_ and FIS scores observed in the PG compared with the CG are consistent with previous studies reporting lower dental anxiety in children exposed to music-based approaches. A recent systematic review and meta-analysis reported that music-based interventions may contribute to lower dental anxiety and fear in pediatric dental patients [15]. Similarly, Janthasila and Keeratisiroj reported lower dental anxiety and fear scores following a music-based intervention in children [27]. However, these studies primarily evaluated passive music listening as an intervention immediately before or during dental treatment. In contrast, the present study examined children with pre-existing, regular piano training as part of their daily lives. Therefore, the findings of the present study should be interpreted within a different context: rather than evaluating the acute effects of music as a distraction technique, this study identified an association between sustained active instrumental training and lower dental anxiety scores.

Evidence from active instrumental engagement provides an additional perspective beyond passive music-listening interventions. Sülün et al. [30] evaluated an 8-week activity-based guitar training program in adolescent relatives of cancer patients aged 11–17 years and reported significant reductions in both state and trait anxiety following the program. In a 2026 systematic review of 20 studies involving 1912 adolescents aged 12–18 years, Lu et al. [31] reported that active forms of musical engagement, including instrument playing, singing, and improvisation, tended to produce more robust and sustained reductions in anxiety than passive music listening. However, to our knowledge, studies specifically examining the association between pre-existing active instrumental training and dental anxiety in children are lacking. Therefore, the present study adds to the existing literature by examining regular, long-term piano training in relation to dental anxiety and perceived stress in children.

Possible pathways that may help explain these associations may involve executive-function and emotion-regulation processes. In children, structured music training has also been associated with executive functions, particularly inhibitory control, working memory, and cognitive flexibility [11]. Active musical engagement has also been linked to emotion-regulation processes [12]. These findings provide a plausible framework for interpreting the associations observed in the present study. Although perceived stress was directly assessed using the PeSSKi, executive functions and emotion-regulation abilities were not specifically measured and should therefore be considered possible explanatory pathways rather than mechanisms demonstrated by the present study.

The significant group × time interactions observed for HR and systolic BP in the present study indicate that changes in these cardiovascular parameters across the dental procedure differed between the PG and CG. HR and systolic BP may reflect acute stress-related autonomic responses [7], and previous studies have reported changes in these parameters in association with music-based interventions during stressful clinical procedures [27,32]. Janthasila and Keeratisiroj reported a significant reduction in systolic BP in children receiving a music-based intervention, while Tan et al. [32] reported in a systematic review and meta-analysis that music interventions may be beneficial in reducing physiological arousal associated with dental anxiety. However, no significant group × time interactions were observed for SpO_2_, diastolic BP, or BT in the present study. Thus, differences in the pattern of change between the PG and CG were observed for HR and systolic BP, but not consistently across all physiological parameters.

Perceived stress is an important factor that may influence children’s behavioral and emotional responses during dental treatment. Responses to dental procedures may be related not only to immediate dental anxiety but also to the child’s general perception of stress and ability to cope with uncertainty [33]. In this study, the lower PeSSKi scores observed in the PG compared with the CG suggest that regular piano training may be associated with lower perceived stress levels in children. PeSSKi scores decreased over time in both groups; however, the group × time interaction was not significant, indicating that the pattern of change was similar in the PG and CG. Thus, the lower PeSSKi scores observed in the PG were present at both assessment times rather than emerging as a differential response to the dental procedure. Previous studies on music interventions have reported associations with lower psychological stress responses and emotion-regulation processes [12,34]. Importantly, PeSSKi assesses perceived stress rather than dental anxiety specifically, and therefore provides complementary information to the MCDAS_f_ and FIS findings. Taken together, these findings suggest that the association with piano-training status extended beyond dental anxiety measures to a broader stress-related construct.

Within the PG, duration of formal piano education was associated with several psychological outcomes. Active instrumental training involves sustained attention, performance control, and emotional self-regulation, characteristics that may be relevant to coping with stressful situations. Toyoshima et al. [35] reported lower stress following piano playing than following other creative art activities, while Kömürcü [36] reported an association between playing a musical instrument and lower vulnerability to stress and more favorable stress-coping skills. In exploratory within-group analyses controlling for age, longer durations of formal piano education remained negatively associated with PeSSKi scores at both T1 and T2, whereas its association with post-treatment MCDAS_f_ was attenuated and did not reach statistical significance (r = −0.315, *p* = 0.051). This attenuation suggests that age may partly contribute to the previously observed association between training duration and post-treatment dental anxiety. In contrast, the associations of weekly formal piano lesson duration with post-treatment MCDAS_f_ and PeSSKi scores remained associated at the nominal *p* < 0.05 level after controlling for age, suggesting that these associations were not fully explained by age differences within the PG. Home piano practice duration was not significantly associated with MCDAS_f_, FIS, or PeSSKi scores. The observed associations with weekly formal piano lesson duration, but not with home practice duration, may partly reflect differences in access to structured instruction and family-related resources rather than training exposure alone. Therefore, given the exploratory nature of these analyses and the potential influence of unmeasured family-related and educational factors, these findings should be interpreted cautiously and should not be considered evidence of a direct effect of piano training exposure.

Neither duration of formal piano education nor weekly formal piano lesson duration was significantly correlated with FIS scores. This difference in findings may partly reflect the measurement characteristics of the instruments. The FIS is a single-item, five-category measure, whereas the MCDAS_f_ and PeSSKi are multi-item instruments assessing dental anxiety and perceived stress, respectively. The more limited scoring range of the FIS may have reduced its sensitivity to subtle associations with piano-training characteristics; however, this explanation remains tentative. Taken together, these findings indicate that selected characteristics of formal piano training were associated with specific dental anxiety and perceived stress measures within the PG, whereas similar associations were not observed for home practice duration or consistently across all assessment measures. Given the exploratory nature of these within-group analyses and the relatively small sample size, these correlations should be interpreted cautiously and require confirmation in larger studies.

The present study has several strengths. To our knowledge, this is among the first studies in pediatric dentistry to examine the association between pre-existing, long-term active instrumental training—specifically regular piano training—and dental anxiety, perceived stress, and physiological responses during dental treatment. This approach extends previous research that has predominantly focused on passive music-listening interventions applied immediately before or during dental procedures [15]. Dental anxiety and stress responses were evaluated using a multidimensional approach that included psychometric, projective, physiological, and perceived stress measures. Furthermore, duration of formal piano education, weekly formal piano lesson duration, and home piano practice duration outside formal lessons were recorded, allowing different dimensions of musical exposure to be examined in relation to anxiety and stress outcomes. All fissure sealant procedures were performed by the same pediatric dentist in the same clinical setting using a standardized clinical protocol. The groups were comparable with respect to age, sex, family income, previous dental experience, DMFT, and PUFA scores, supporting comparability across several measured baseline characteristics. Also, all participants completed the planned procedure and both assessment time points, with no post-enrollment exclusions or incomplete treatments.

Nevertheless, several limitations should be considered when interpreting the findings. Although participants were randomly selected from the eligible pools, group membership was determined by pre-existing piano training status rather than by random allocation. Therefore, reverse causation and selection bias cannot be excluded. The lower PeSSKi scores observed in the PG already at T1 indicate that between-group differences in perceived stress were present before the dental procedure. Because T1 represented the pre-treatment assessment rather than a pre-piano-training baseline, this difference may be consistent with an association between long-term piano training and lower perceived stress; however, pre-existing differences between the groups cannot be excluded and should be considered in relation to potential selection bias. Children who received regular piano training may have differed from controls in pre-existing family, educational, socioeconomic, or individual characteristics that were not fully captured by the measured variables and therefore could not be completely accounted for through statistical adjustment.

Children with lower pre-existing anxiety, better emotional self-regulation, or particular temperamental characteristics may have been more likely to initiate and continue piano training, and these characteristics could also be independently associated with lower anxiety and stress responses during dental treatment. Baseline temperament and general, non-dental anxiety were not assessed using validated instruments and therefore could not be included as covariates. Although the baseline-adjusted analyses accounted for pre-treatment outcome scores and parental education, unmeasured individual, socioeconomic, sociocultural, and family-level factors may still have contributed to the observed associations. Thus, residual confounding remains possible.

Although ethical approval was obtained before participant enrollment, registration on ClinicalTrials.gov was completed retrospectively after participant enrollment and data collection had ended due to administrative and documentation-related procedures. This retrospective registration should be considered when evaluating the methodological rigor of the study.

Another limitation concerns the effect size used in the a priori sample size calculation. The effect size (d = 0.85) was derived from a pharmacological randomized controlled trial involving preschool children under general anesthesia and may therefore have overestimated the effect expected in the present study. Consequently, the study may have had limited statistical power to detect smaller effects, particularly in the adjusted and within-group analyses, and nonsignificant findings from these analyses should be interpreted with caution.

Single-center design may limit the generalizability of the findings. Although formal training duration, weekly lesson duration, and home practice duration were examined, these quantitative measures may not fully capture overall musical exposure. Other potentially relevant dimensions, including practice quality, day-to-day consistency, level of engagement, musical proficiency, and individual aptitude, were not systematically assessed. Finally, only piano training was investigated; therefore, future multicenter, prospective longitudinal studies including different musical instruments, broader psychological variables, and more comprehensive measures of musical exposure are needed to better characterize these associations.

## 5. Conclusions

In conclusion, pre-treatment MCDAS_f_ scores did not differ between the groups, whereas lower post-treatment MCDAS_f_ scores were observed in the PG. In contrast, PeSSKi scores were already lower in the PG before treatment and remained lower after treatment, indicating a between-group difference in perceived stress that was already present before the dental procedure. This finding is consistent with the possibility that children receiving regular piano training may have lower overall levels of perceived stress. Regular piano training was also associated with lower FIS scores and differences in selected physiological responses during pit and fissure sealant application. Exploratory within-group analyses controlling for age showed that longer formal piano education was associated with lower perceived stress, while greater weekly formal piano lesson duration was associated with lower post-treatment dental anxiety and perceived stress. Because *p* values were unadjusted for multiple comparisons, these findings should be interpreted cautiously. Given the observational design, prospective longitudinal studies are needed to clarify the temporal direction of these associations and to determine whether sustained instrumental training has a causal influence on children’s emotional responses during dental care.

## Figures and Tables

**Table 1:** Generalized estimating equation analyses of MCDAS_f_, FIS, and PeSSKi scores according to group, time, parental education level, and group × time interaction.

Variables	Time	Groups		Total	Group	Time	Parental Education Level	Group × Time
		Piano	Control					
**MCDAS_f_**	T1	20.40 ± 4.35 ^A^	21.18 ± 3.39 ^A^	20.79 ± 3.89	7.056: **0.008**	244.107: <**0.001**	2.292: 0.318	16.162: <**0.001**
	T2	15.03 ± 3.27 ^C^	18 ± 3.74 ^B^	16.51 ± 3.8				
	Total	17.71 ± 4.68	19.59 ± 3.89					
**FIS**	T1	1.9 ± 0.74	2.13 ± 0.69	2.01 ± 0.72	5.675: **0.017**	88.235: <**0.001**	1.04: 0.594	0.392: 0.531
	T2	1.1 ± 0.3	1.43 ± 0.5	1.26 ± 0.44				
	Total	1.5 ± 0.69	1.78 ± 0.69					
**PeSSKi**	T1	20.78 ± 5.28	31.48 ± 3.08	26.13 ± 6.89	142.009: <**0.001**	78.903: <**0.001**	5.321: 0.070	0.280: 0.597
	T2	18.78 ± 5.27	29.7 ± 3.37	24.24 ± 7.04				
	Total	19.78 ± 5.34	30.59 ± 3.33					

Generalized Estimating Equations (GEE); values are presented as mean ± standard deviation. Superscript letters A–C indicate post hoc pairwise comparisons; values sharing the same letter do not differ significantly. T1, before dental treatment; T2, after dental treatment; MCDAS_f_, Modified Child Dental Anxiety Scale–faces version; FIS, Facial Image Scale; PeSSKi, Perceived Stress Scale for Kids. Bold values indicate statistical significance (*p* < 0.05).

**Table 2:** Baseline-adjusted ANCOVA results for post-treatment MCDAS_f_, FIS, and PeSSKi scores.

Variables		Test Statistic	*p*	Partial eta Squared [95% CI]
**Post-treatment MCDAS_f_**	Group	35.648	**<0.001** ^z^	0.319 [0.140–0.518]
	Baseline MCDAS_f_	134.167	**<0.001** ^z^	0.638 [0.495–0.761]
	Parental education level	9.597	**0.003** ^z^	0.112 [0.023–0.273]
**Post-treatment FIS**	Group	5.619	**0.020** ^y^	0.073 [0.002–0.240]
	Baseline FIS	6.287	**0.014** ^y^	0.080 [0.006–0.241]
	Parental education level	0.867	0.355 ^y^	0.017 [0.000–0.140]
**Post-treatment PeSSKi**	Group	4.266	**0.042** ^x^	0.059 [0.001–0.236]
	Baseline PeSSKi	352.575	<**0.001** ^x^	0.819 [0.746–0.886]
	Parental education level	5.324	**0.024** ^x^	0.055 [0.001–0.210]

^x^ HC3-corrected robust ANCOVA; ^y^ bootstrapped ANCOVA with 500 bootstrap samples; ^z^ one-way ANCOVA. For each outcome, the corresponding pre-treatment score was included in the model as a covariate. Effect sizes are presented as partial eta squared with 95% confidence intervals (CIs). MCDAS_f_, Modified Child Dental Anxiety Scale—faces version; FIS, Facial Image Scale; PeSSKi, Perceived Stress Scale for Kids. Bold values indicate statistical significance (*p* < 0.05).

**Table 3:** Comparison of physiological parameters according to group and time.

Variables	Groups	Time		Total	Group t *p*	Time t *p*	Group × Time t *p*
		T1	T2				
**HR**	Piano	89.53 ± 9.81 ^A^	85.45 ± 8.67 ^B^	87.49 ± 9.43	0.076; 0.783	4.948; **0.026**	23.862; **<0.001** ^x^
	Control	87.3 ± 10.28 ^AB^	88.83 ± 10.33 ^AB^	88.06 ± 10.27			
	Total	88.41 ± 10.05	87.14 ± 9.63				
**SpO_2_**	Piano	97 (93: 99)	97 (92: 100)	97 (92: 100) /76.662	0.004; 0.951	4.793; **0.032**	2.359; 0.129 ^y^
	Control	97 (95: 99)	97 (94: 99)	97 (94: 99) /84.337			
	Total	97 (93: 99)	97 (92: 100)				
**Systolic BP**	Piano	104.35 ± 8.17 ^A^	101.65 ± 8.02 ^B^	103 ± 8.16	0.692; 0.406	2.755; 0.097	4.818; **0.028** ^x^
	Control	104.23 ± 8.2 ^AB^	104.6 ± 8.86 ^AB^	104.41 ± 8.49			
	Total	104.29 ± 8.14	103.13 ± 8.53				
**Diastolic BP**	Piano	66 (59: 84)	65 (57: 90)	65.5 (57: 90)	0.651; 0.422	0.489; 0.487	1.678; 0.199 ^y^
	Control	66 (56: 82)	69 (57: 84)	67.5 (56: 84)			
	Total	66 (56: 84)	68 (57: 90)				
**BT**	Piano	36.3 (36.1: 36.5)	36.3 (36: 36.7)	36.3 (36: 36.5)	0.000; 0.994	0.000; 0.836	0.000; 0.258 ^y^
	Control	36.3 (36: 36.5)	36.3 (36: 37)	36.3 (36: 37)			
	Total	36.3 (36: 36.7)	36.3 (36: 37)				

^x^ Generalized estimating equations (GEE); ^y^ Aligned Rank Transform (ART) mixed ANOVA; t, values in the test statistic columns are presented as test statistic; *p* value, data are presented as mean ± standard deviation or median (minimum–maximum); superscript letters indicate post hoc comparisons, and groups sharing the same letter did not differ significantly; HR, heart rate; BP, blood pressure; SpO_2_, peripheral oxygen saturation; BT, body temperature; T1, before dental treatment; T2, after dental treatment. Bold values indicate statistical significance (*p* < 0.05); Superscript letters A and B indicate post hoc pairwise comparisons; values sharing the same superscript letter are not significantly different, whereas values with different superscript letters are significantly different (*p* < 0.05).

**Table 4:** Partial correlations between piano training characteristics and dental anxiety and perceived stress measures at T1 and T2, controlling for age.

Variables	Measurement Time	Duration of Formal Piano Education (Months)		Weekly Formal Piano Lesson Duration (Hours/Week)		Home Piano Practice Duration Outside Formal Lessons (Hours/Week)	
		r	*p*	r	*p*	r	*p*
**MCDAS_f_**	T1	−0.252	0.121	−0.250	0.124	−0.293	0.070
	T2	−0.315	0.051	−0.461	**0.003**	−0.280	0.084
**PeSSKi**	T1	−0.354	**0.027**	−0.307	0.057	−0.124	0.452
	T2	−0.376	**0.018**	−0.419	**0.008**	−0.231	0.156
**FIS**	T1	−0.212	0.195	−0.212	0.196	−0.161	0.327
	T2	0.005	0.976	0.095	0.566	−0.201	0.220

r, partial correlation coefficient calculated while controlling for age; T1, before dental treatment; T2, after dental treatment; MCDASf, Modified Child Dental Anxiety Scale—faces version; FIS, Facial Image Scale; PeSSKi, Perceived Stress Scale for Kids; Bolded values indicate statistical significance (*p* < 0.05).

## Data Availability

The data that support the findings of this study are available from the corresponding author upon reasonable request.

## Supplementary Materials

The following supporting information can be downloaded at https://www.mdpi.com/article/10.3390/children13101367/s1, Table S1: Descriptive statistics of MCDAS_f_, FIS, and PeSSKi scores according to parental education level; Table S2: Descriptive statistics of post-treatment MCDAS_f_, FIS, and PeSSKi scores according to group and parental education level.
