# Supplementary material for: Association of Regular Piano Training with Dental Anxiety and Stress in Children Undergoing Pit and Fissure Sealant Application: A Cross-Sectional Study
Source: Children (Basel). 2026 Oct 8;13(10):1367. doi: 10.3390/children13101367 (PMC13703332; doi:10.3390/children13101367)
Supplement: Supplementary file 1 [file children-13-01367-s001.zip › children-4565529-supplementary.pdf]

Table S1. Descriptive statistics of MCDAS$_f$, FIS, and PeSSKi scores according to parental education level.

	Middle school			High school			University			Total	
	T1	T2	Total	T1	T2	Total	T1	T2	Total	T1	T2
**MCDAS$_f$**	22.4 ± 3.44	17.2 ± 2.59	19.8 ± 3.97	20.29 ± 3.51	15.94 ± 3.82	18.12 ± 4.23	20.79 ± 4.05	16.62 ± 3.91	18.71 ± 4.48	20.79 ± 3.89	16.51 ± 3.8
**FIS**	2 ± 1	1.2 ± 0.45	1.6 ± 0.84	2 ± 0.61	1.59 ± 0.51	1.79 ± 0.59	2.02 ± 0.74	1.17 ± 0.38	1.59 ± 0.72	2.01 ± 0.72	1.26 ± 0.44
**PeSSKi**	30.2 ± 2.17	27.4 ± 3.05	28.8 ± 2.9	28.47 ± 5.95	26.24 ± 6.22	27.35 ± 6.1	25.09 ± 7.16	23.38 ± 7.37	24.23 ± 7.29	26.13 ± 6.89	24.24 ± 7.04

Values are presented as mean ± standard deviation. T1, before dental treatment; T2, after dental treatment; MCDAS$_f$, Modified Child Dental Anxiety Scale–faces version; FIS, Facial Image Scale; PeSSKi, Perceived Stress Scale for Children.

Table S2. Descriptive statistics of post-treatment MCDAS$_f$, FIS, and PeSSKi scores according to group and parental education level.

	Post-treatment MCDAS$_f$	Post-treatment FIS	Post-treatment PeSSKi
**Group**			
**Piano**	15.03 ± 3.27	1.10 ± 0.30	18.78 ± 5.27
**Control**	18.00 ± 3.74	1.43 ± 0.50	29.70 ± 3.37
**Parental education level**			
**Middle school**	17.20 ± 2.59	1.20 ± 0.45	27.40 ± 3.05
**High school**	15.94 ± 3.82	1.59 ± 0.51	26.24 ± 6.22
**University**	16.62 ± 3.91	1.17 ± 0.38	23.38 ± 7.37

Values are presented as mean ± standard deviation. MCDAS$_f$, Modified Child Dental Anxiety Scale–faces version; FIS, Facial Image Scale; PeSSKi, Perceived Stress Scale for Children.
